# Duration of veno-arterial extracorporeal life support (VA ECMO) and outcome: an analysis of the Extracorporeal Life Support Organization (ELSO) registry

**DOI:** 10.1186/s13054-017-1633-1

**Published:** 2017-03-06

**Authors:** Myles Smith, Alexander Vukomanovic, Daniel Brodie, Ravi Thiagarajan, Peter Rycus, Hergen Buscher

**Affiliations:** 1 Department of Intensive Care Medicine, St Vincent’s Hospital, Sydney, NSW Australia; 2 Extracorporeal Life Support Organization (ELSO), Ann Arbor, MI USA; 3 University of New South Wales, Sydney, NSW Australia; 4 Columbia University Medical Center/NewYork-Presbyterian Hospital, New York, NW USA

**Keywords:** Extracorporeal membrane oxygenation, Extracorporeal Life Support Organization, Refractory shock, Treatment duration, Survival, Outcomes

## Abstract

**Background:**

Veno-arterial extracorporeal membrane oxygenation (VA ECMO) is an effective rescue therapy for severe cardiorespiratory failure, but morbidity and mortality are high. We hypothesised that survival decreases with longer VA ECMO treatment. We examined the Extracorporeal Life Support Organization (ELSO) registry for a relationship between VA ECMO duration and in-hospital mortality, and covariates including indication for support.

**Methods:**

All VA runs from the ELSO database from 2002 to 2012 were extracted. Multiple runs and non-VA runs were excluded. Runs were categorized into diagnostic groups. Logistic regression for analysis of the effect of duration on outcome, and multivariate regression for diagnosis and other baseline factors were performed. Non-linear models including piecewise logistic models were fitted.

**Results:**

There were 2699 runs analysed over 14,747 days. Logistic regression analysis of the effect of duration on outcome, and multivariate regression analysis of diagnosis and other baseline factors were performed. In-hospital survival was 41.4% (95% CI 39.6–43.3). 75% of patients were supported for less than 1 week and 96% for less than 3 weeks. Median duration (4 days IQR 2.0–6.8) was greater in survivors (4.1 (IQR 2.5–6.7) vs 3.8 (IQR 1.7–7.0) *p* = 0.002). The final multivariate model demonstrated increasing survival to day 4 (OR 1.53 (95% CI 1.37–1.71) *p* < 0.001), decreasing from day 4 to 12 (OR 0.86 (95% CI 0.81–0.91), *p* < 0.001) with no significant change thereafter (OR 0.98 (95% CI 0.94–1.02), *p* = 0.400).

**Conclusions:**

ECMO for 4 days or less is associated with higher mortality, likely reflecting early treatment failure. Survival is highest when patients are weaned on the fourth day of ECMO but likely decreases into the second week. While this does not suggest weaning at this point will produce better outcomes, it does reflect the likely time course of ECMO as a bridge in severe shock. Patients with some underlying conditions (like myocarditis and heart transplantation) achieve better outcomes despite longer support duration. These findings merit prospective study for the development of prognostic models and weaning strategies.

## Background

Veno-arterial extracorporeal membrane oxygenation (VA ECMO) is an effective rescue therapy for severe cardiorespiratory failure, but morbidity and mortality are high [1]. As opposed to veno-venous (VV ECMO) therapy for severe respiratory failure, VA ECMO provides partial or complete haemodynamic support in the case of severe cardiovascular compromise. Use of this treatment modality is expanding internationally, with increasing numbers reported in international databases, most notably the Extracorporeal Life Support Organization (ELSO) registry with 7850 adult cardiac VA ECMO cases to date, with an overall survival to discharge of 41% [2].

Prognostic models for survival in VA ECMO treatment have been developed, such as the survival after veno-arterial ECMO (SAVE) score [3], based on pre-ECMO patient and disease factors. As treatment becomes more sophisticated, and centres have more experience, prolonged therapy may become more acceptable and common. There are some survivors of long-term support [4], but the relationship between treatment duration and survival is unclear.

Some cohort studies of mixed VA ECMO patients have identified similar treatment duration in survivors and non-survivors on VA ECMO [5]. In terms of other support types, studies in veno-venous ECMO have found no decrease in survival with long-term support [6, 7]. However, in another study of post-cardiac surgery VA ECMO support in children the odds of mortality (OR 1.12) increased per day of treatment [8]. Analysis of children treated with VA ECMO for more than 14 days in the ELSO registry showed decreased survival with longer treatment duration [9]. Given the significant incidence of haemorrhagic, neurologic and septic complications of therapy, prolonged therapy may lead to increased mortality, with one analysis showing significantly increased rates of infection with increased support time [10]. We hypothesised that analysis of the ELSO registry would identify decreasing survival with longer runs on VA ECMO.

Given anticipated differences in outcome and treatment duration between medical and post-surgical indications for ECMO, we hypothesised that the relationship between duration of ECMO and survival may vary depending on the underlying indication. We examined the ELSO database for evidence of a relationship between VA ECMO treatment duration and survival to hospital discharge. We aimed to examine the effect of available covariates and interactions with the indication for ECMO support.

## Methods

A retrospective study of the ELSO registry data was conducted. This voluntary database collects baseline data and outcome data on patients undergoing ECMO treatment in participating centres, with a total of 232 centres contributing up to 2012 [2]. Data collected include age, sex, weight, surgical procedures performed, primary and other diagnoses, discharge location, basic ventilation data, haemodynamic variables and arterial blood gas results. All adult VA ECMO runs from the ELSO database from 2002 to 2012 were extracted. This excludes paediatric and neonatal patients, patients undergoing extracorporeal cardiopulmonary resuscitation (E-CPR), and patients on veno-venous (respiratory support). Multiple runs and non-VA runs were then excluded. Runs with missing duration data were excluded. There was no imputation for missing data.

Primary diagnoses were categorised by two reviewers into predefined post-surgical groups (post-transplant, post-ventricular assist device (VAD) and other cardiac surgery), and medical groups (coronary artery disease, myocarditis, chronic structural, other cardiac and other non-cardiac). There were 130 ECMO runs without diagnostic data.

Treatment duration and other continuous variables were examined for normality using the Shapiro-Wilk test, and in particular, treatment duration was found to be neither normally distributed, nor log-normally distributed. Normally distributed variables were compared using the Student *t* test, while non-normally distributed variables were compared using the Kruskal-Wallis test. Categorical variables were examined using the Fisher exact test. Statistical significance was determined at the level of *p* < 0.05.

Survival analysis was then used to examine treatment duration as a continuous outcome variable. This was done primarily to examine any pattern in changing survival with time. Given that two mutually exclusive events could occur at any time on ECMO (successful weaning with survival, or death), a competing risks [11] approach was used to plot these two discontinuation states over time. Hazard function estimates for survival to hospital discharge were then examined for a continuous relationship between time on ECMO and probability of survival.

Regression models were then developed to analyse the effect of treatment duration on survival. Logistic regression was performed to analyse the effect of treatment duration on outcome, and multivariate regression was performed to analyse the effect of diagnostic category and other baseline factors collected in the ELSO database. Given the non-monotonic nature of the hazard function demonstrated with survival analysis, non-linear models were used to model non-linear relationships between treatment duration and survival. These included piecewise logistic regression with different break points for duration near the observed peak of survival, and polynomials. Piecewise break point selection was performed algorithmically as per Muggeo [12] and with manually selected break points near local extrema of survival. Models were compared for maximal goodness of fit.

In order to fairly compare models in the presence of missing data for some covariates, the dataset was restricted to the subset of clinically likely meaningful covariates (age, sex, weight, time from intubation, arterial blood gas (ABG) variables, simple haemodynamic variables, year of treatment and diagnostic category). Analyses were performed using R software (R Foundation for Statistical Computing, Vienna, Austria; 2014), including the use of the packages *cmprsk* [13], *bshazard* and *segmented* [12].

## Results

**Fig. 1:**
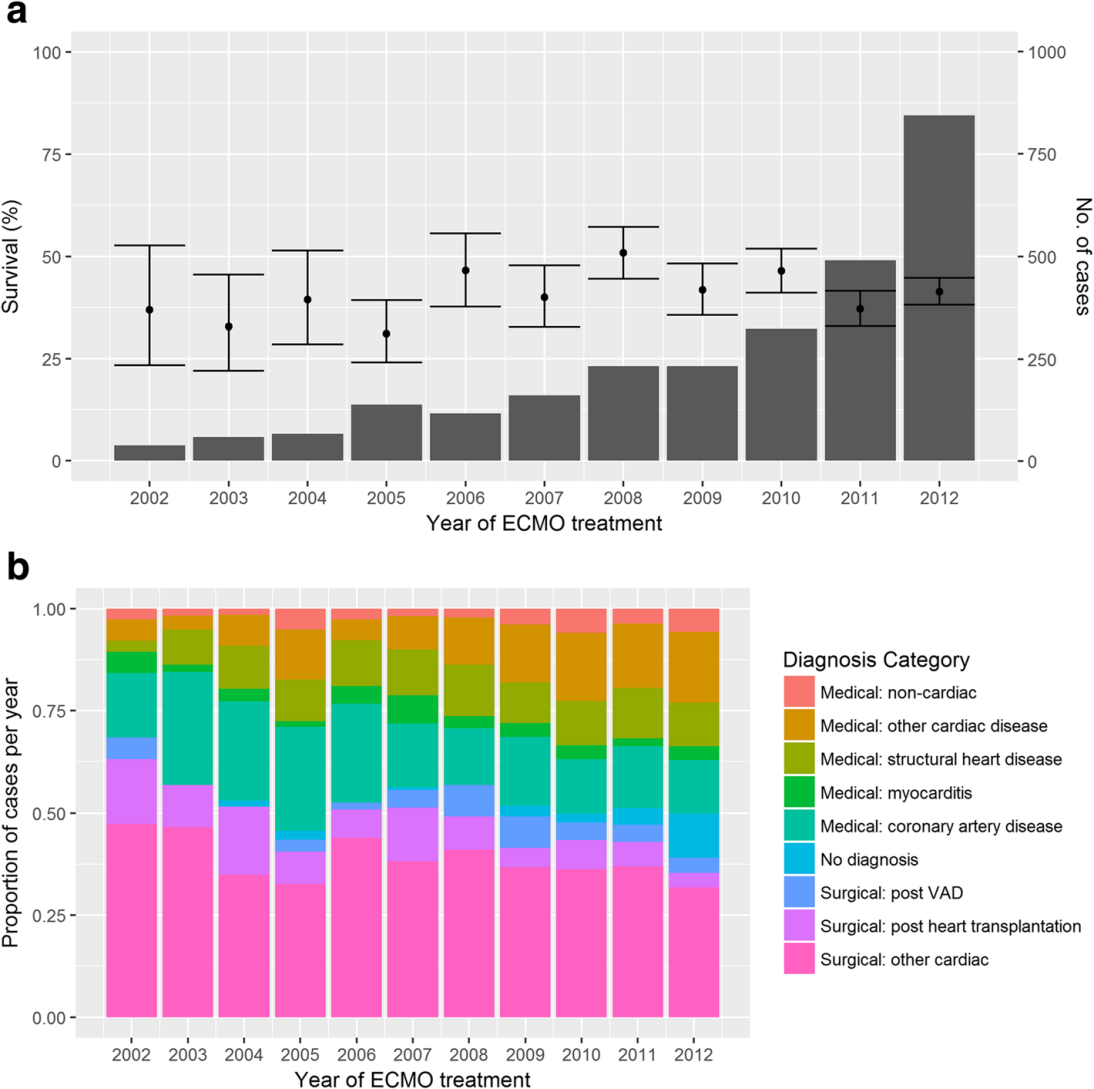
Trends in survival and diagnosis by year of extracorporeal membrane oxygenation (*ECMO*) treatment. **a** Survival by year of ECMO treatment, with total number of patients treated that year, and 95% confidence intervals for survival. **b** Diagnostic groups by year of ECMO treatment. *VAD* ventricular assist device

In total, 2699 runs over 14,747 days of VA ECMO were analysed. Summary characteristics of the data are presented, and compared between groups in Table 1. Overall survival to hospital discharge was 41.4% (95% CI 39.6–43.3). The duration of ECMO support varied (0–87 days), with a long tail of long-term support: 75% of patients were supported for less than 1 week and 96% were supported for less than 3 weeks. Median duration (4 days IQR (2.0–6.8)) was greater in survivors (4.1 (IQR 2.5–6.7) vs 3.8 (IQR 1.7–7.0) *p* = 0.002). The number of patients treated increased from 38 in 2002 to 846 in 2012. There was significant variation in survival between years ranging from 31.2% in 2005 to 50.9% in 2008 (*p* = 0.003), but no clear trend (Fig. 1). The proportion of diagnoses varied between years, with a trend towards increasing medical indications (Kendall’s tau 0.477, *p* = 0.042 for proportion with medical indications of the total cases per year).

**Table 1:** Baseline characteristics and comparison of survivors and non-survivors

Variable	Overall	Died	Survived	*P* value
Number of patients	2699	1582	1117	
Days of ECMO (median (IQR))	4.00 (1.96, 6.83)	3.83 (1.67, 7.00)	4.13 (2.46, 6.67)	0.002
Age (years) (median (IQR))	54.0 (42.0, 63.0)	56.0 (44.0, 65.0)	52.0 (38.0, 61.0)	<0.001
Sex (%)				0.341
Female	862 (31.9)	522 (33.0)	340 (30.4)	
Male	1815 (67.2)	1048 (66.2)	767 (68.7)	
Weight (kg) (median (IQR))	75.0 (64.0, 88.9)	76.0 (63.7, 90.0)	75.0 (64.1, 86.0)	0.138
Intubation to ECMO (median (IQR))^a^	10.0 (4.0, 25.0)	12.0 (5.0, 31.0)	8.5 (3.0, 22.0)	<0.001
pH (median (IQR))	7.31 (7.21, 7.40)	7.30 (7.19, 7.39)	7.32 (7.23, 7.40)	0.001
Bicarbonate (mmol/l) (median (IQR))	20.0 (16.3, 23.1)	19.2 (15.4, 23.0)	20.6 (17.3, 24.0)	<0.001
MAP (mmHg) (median (IQR))	59 (49, 70)	57 (47, 68)	62 (51, 72)	<0.001

^a^Number of hours from endotracheal intubation to initiation of extracorporeal membrane oxygenation (ECMO). Sex not specified in 22 cases. pH, bicarbonate and mean arterial blood pressure (MAP) were all measured prior to initiation of ECMO. *IQR* interquartile range

**Fig. 2:**
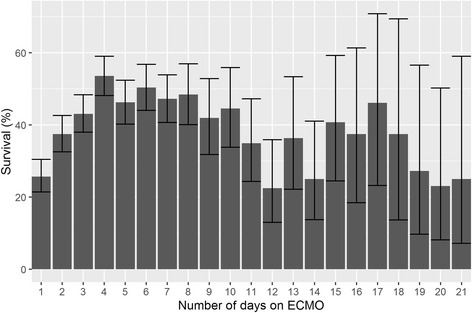
Survival by day of extracorporeal membrane oxygenation (*ECMO*) discontinuation. *Errors bars* show 95% binomial confidence interval for survival

**Fig. 3:**
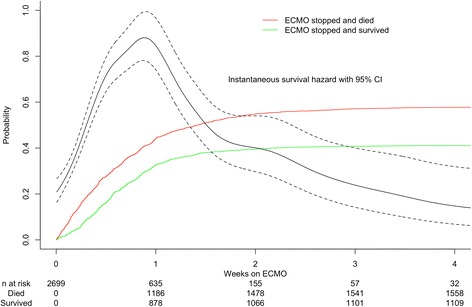
Estimated survival hazard in the first 4 weeks, with cumulative incidence of survival and mortality. *Coloured curves* show cumulative incidence of survival after cessation of extracorporeal membrane oxygenation (*ECMO*) (*green*), and mortality after ECMO (*red*), by duration of treatment. Instantaneous hazard function estimate for survival plotted in *black* with 95% confidence interval. Numbers of patients at risk and who survived or died are shown per week below the chart

When calculated by day of ECMO discontinuation (Fig. 2), survival increased from 25.7% on the first day (95% CI 21.4– 30.5) and peaked on day 4 at 53.6% (95% CI 48.1–59.0). Hazard function for survival was not constant, with a biphasic distribution peaking around day 7 (Fig. 3). On inspection, survival appeared to decrease after this point, but the confidence intervals were wide due to decreasing numbers of patients at risk.

Univariate logistic regression analysis of the effect of ECMO duration on survival was best fit using segmented regression with break points at day 4 and day 12. There was no improvement in fit with other break points or polynomial models. There was increasing survival per day of ECMO prior to day 4 (odds ratio (OR) 1.39 (95% CI 1.29–1.50), *p* < 0.001) and then decreasing survival from day 4 to 12 (OR 0.89 (95% CI 0.86–0.93), *p* < 0.001). From day 12 onwards there was no significant change (OR 0.99 (95% CI 0.96–1.01), *p* = 0.356).

On multivariate regression analysis with adjustment for the covariates available there were similar trends of survival by day, increasing to day 4 (OR 1.53 (95% CI 1.37–1.71), *p* < 0.001), and decreasing from day 4 to 12 (OR 0.86 (95% CI 0.81–0.91), *p* < 0.001) with no significant change thereafter (OR 0.98 (95% CI 0.94–1.02), *p* = 0.400). The significant covariates remaining in the final model were diagnostic category (*p* = 0.008), age (*p* = <0.001), pH (*p* = 0.005), mean arterial pressure (*p* < 0.001), and time from endotracheal intubation to initiation of ECMO (*p* = 0.049).

There were statistically significant differences in survival (*p* < 0.001) with better survival in myocarditis (64.4%) and post-heart transplantation (57.1%), and poorer survival in other medical cardiac disease (38.4%) and other surgical cardiac disease (35.9%) (Table 2). There were also differences in treatment duration by diagnosis (*p* < 0.001) with longer duration also in myocarditis and heart transplantation, and shorter duration post VAD. However, there was no significant interaction between diagnosis and treatment duration in terms of effect on survival in any model.

**Table 2:** Duration of treatment, overall hospital survival and survival by week of ECMO termination

				Overall survival (95% confidence interval)	Survival at weeks 1, 2 and 3		
Clinical category	Number	Median duration (h)	IQR		Week 1	Week 2	Week 3+
Medical: non-cardiac	115	87	31–170	49.6% (40.6–58.6)	53.5% (43.0–63.7)	42.8% (24.5–63.5)	25.0% (7.1–59.1)
Medical: other cardiac disease	383	96	48–162	38.4% (33.6–43.3)	37.0% (31.7–42.7)	42.0% (31.1–53.8)	47.1% (26.2–69.0)
Medical: structural heart disease	296	117	58–192	44.3% (38.7–50.0)	43.1% (36.4–50.0)	50.7% (39.3–62.0)	34.8% (18.8–55.1)
Medical: myocarditis	87	154	96–230	64.4% (53.9–73.6)	70.6% (57.0–81.3)	62.5% (42.7–78.8)	41.7% (19.3–68.0)
Medical: coronary artery disease	424	109	51–177	40.1% (36.0–45.3)	40.6% (35.3–46.2)	42.2% (32.1–52.9)	35.5% (21.1–53.1)
Surgical: post VAD	117	68	33–122	41.9% (33.3–50.9)	41.0% (32.0–50.5)	44.4% (18.9–73.3)	66.7% (20.8–93.9)
Surgical: post heart transplantation	175	108	66–173	57.1% (49.7–64.2)	60.0% (51.4–68.0)	52.9% (36.7–68.6)	36.3% (15.2–64.6)
Surgical: other cardiac	972	87	41–146	35.9% (33.0–39.0)	38.7% (35.3–42.1)	25.3% (19.0–33.0)	20.0% (10.5–34.8)
Overall	2699	96	47–164	41.4% (39.5–43.3)	42.5% (40.4–44.7)	39.2% (34.9–43.6)	32.9% (26.0–40.6)

*IQR* interquartile range, *VAD* ventricular assist device

**Fig. 4:**
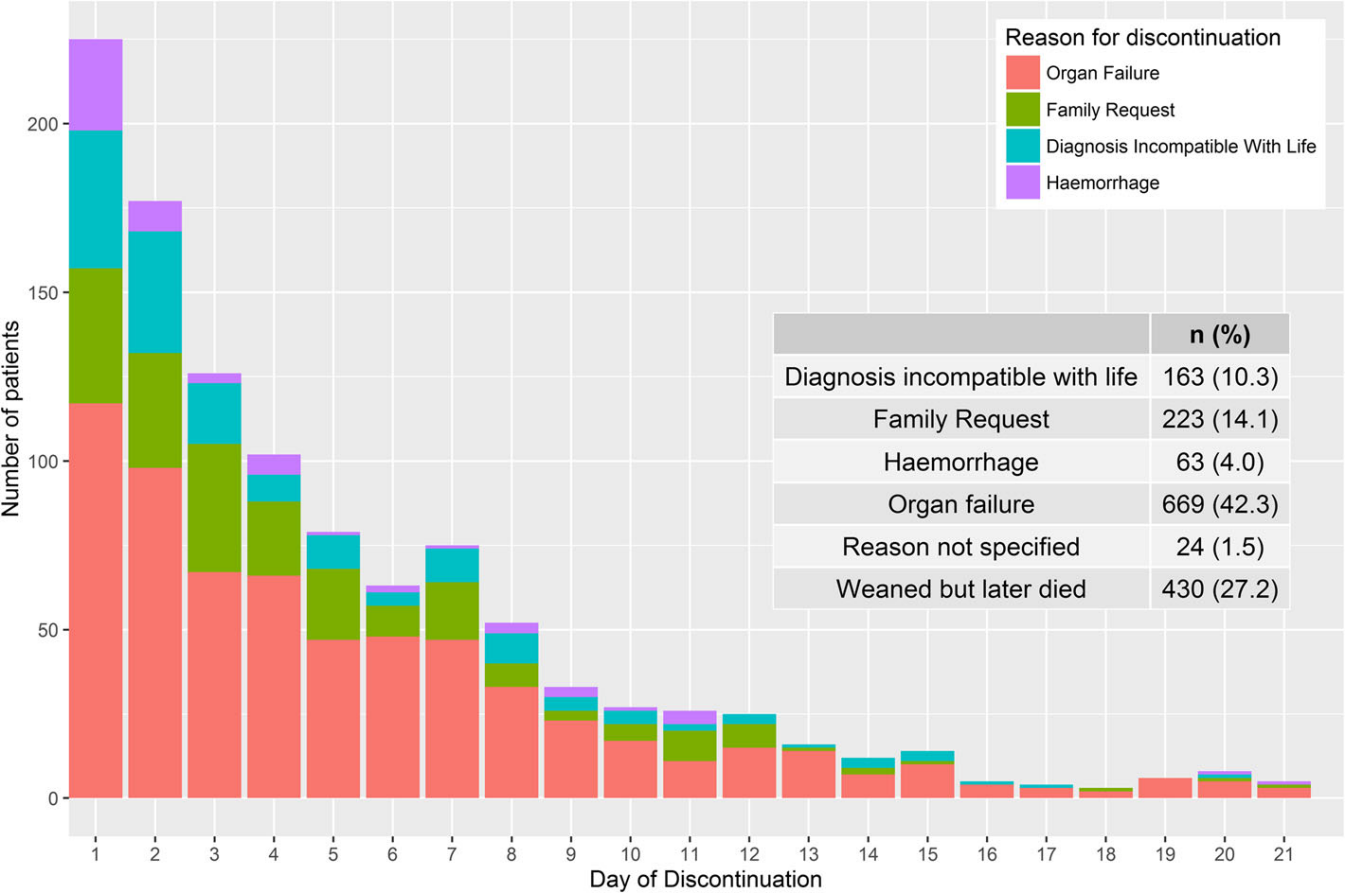
Reasons for discontinuation and death by extracorporeal membrane oxygentation (ECMO) duration. Reasons for discontinuation of ECMO, and death according to ECMO duration are presented. *Colour of bars* indicates reason for discontinuation (*red* organ failure, *green* family request, *blue* diagnosis incompatible with life, *purple* haemorrhage). Overall outcomes and reasons for discontinuation are summarised

Amongst non-survivors, 27% were successfully weaned from ECMO but died prior to discharge. The commonest reason for discontinuation was organ failure, followed by family request, with similar rates during treatment (Fig. 4) other than a comparatively high 7.7% (95% CI 5.3–10.9) mortality rate due to haemorrhage on day 1.

## Discussion

Our analysis of the VA ECMO cases within the ELSO registry demonstrates that mortality varies with the duration of ECMO treatment, but in a complex way. We anticipated that survival would steadily decrease with treatment duration as progression of disease and treatment complications added to cumulative mortality, as suggested previously [8]. However, we found that survival in fact increases in the first few days, peaking mid-week in the first week of treatment. Inspection of the estimated survival hazard function and examination of the regression models fitted suggest that survival decreased significantly up to day 12. After this second change point, there was no evidence of change in survival (that is, the confidence intervals of the slope included zero), or significant change points past this point that added to model fit.

While this does not provide evidence for a point of “futility” beyond which treatment has very low survival, it does provide an insight into the “natural history” of VA ECMO treatment. In particular, this analysis does not suggest that weaning should occur on a particular day in order to maximise survival. Instead, it reflects the role of ECMO as a bridge, whereby the duration of treatment that is required and is sustainable depends on the underlying disease process. Mortality is especially high in the first few days, but patients who survive this period are more likely to then survive when their condition is such that they are weaned in the first week.

Comparison of reasons for discontinuation in this early period suggested that fatal haemorrhage was disproportionately represented in the first day of treatment, whereas organ failure and family request for discontinuation were more evenly spread (Fig. 4). Haemorrhage is well-documented as a common complication [14] of VA ECMO treatment that is associated with increased mortality [15], and this suggests that this danger may be highest in the initiation phase of treatment, at least in cases of severe illness. However, the commonest reasons for discontinuation in this early period were still organ failure and a diagnosis incompatible with life, suggesting that treatment failure predominates over treatment complications during this phase in non-survivors.

In longer ECMO treatment past one week, survival appears to decrease again to near the mean survival, with corresponding relative increases in discontinuation due to organ failure. After three weeks 96% of patients have been weaned successfully or have died, and analysis of data on this “tail” of patients on long-term VA ECMO did not suggest positive or negative correlation between treatment duration and survival. This is similar to a recent analysis from the ELSO registry of the survival fraction in prolonged VV ECMO therapy [16]. This may be due to a balance between increased risk with cumulative exposure to the risks of disease progression and treatment complications (for instance intracranial haemorrhage [17] or infection [10]), and the selection of patients perceived to be more likely to survive for longer treatment. There is also an inherent bias in treatment duration based on clinician decision-making due to arbitrary limits on the amount of time that ECMO is offered. Finally, a trend in survival of patients on long-term ECMO may not be detected due to the relatively small number of patients remaining, and consequently insufficient statistical power.

These results are relevant to the clinical application of VA ECMO, in that they highlight the high mortality rate in the first few days compared to the relatively good outcomes in those that can be supported past this point, although it is unlikely that longer runs themselves are the reason for survival. At the same time, they provide some support to the use of long-term VA ECMO support, in that survival does decrease below the overall mean but does not inevitably have a downward trend with prolonged treatment. This is particularly relevant as the use of ECMO becomes more prevalent, where longer runs may become more common.

In the dataset analysed there was weak correlation between more recent years and increased ECMO duration (Kendall’s tau 0.045, *p* = 0.001), and certainly increasing numbers of patients treated for longer than two weeks, with only 2 of 38 patients in 2002 increasing to 53 of 846 patients in 2012. For example, in recent reports of increased use of VA ECMO as a “bridge to recovery” in pulmonary hypertension in the face of organ scarcity, the mean duration of support was 7.8 days in survivors and 19.3 days in non-survivors [18].

VA ECMO is also increasingly reported to facilitate transplantation of “marginal” organs [19], or for novel indications such as septic shock [20–23]. New approaches to vascular access may also allow longer therapy; using subclavian access a median duration of 7 days was reported with advantages in ambulation and vascular complications [24]. In this dataset, there were larger numbers of outliers with very long support times in the more recent year groups in particular. Interestingly, in one of the largest individual centre datasets comparison of the first 1000 and second 1000 patients indicated decreased support times but correspondingly increased survival, although there are numerous confounders in the comparison between the earlier and more recent sets of patients [25].

Treatment was continued for less than 48 hours in 25.2% (95% CI 23.5–26.8) of patients and the mortality rate was high (69.7% (95% CI 66.1–73.0)). In Germany Karagiannidis et al. [26] identified a similarly high mortality rate in a mixed cohort of adults and paediatric patients, with an even higher incidence (more than one third) of short VA ECMO treatment times. As was pointed out in an editorial [27], the key to avoiding these early mortalities is careful patient selection and avoidance of treatment-related complications. However, an intervention often instituted as a rescue treatment to avoid death, will (by definition) have a high early failure rate. Recognition of this is important to avoid unnecessarily prolonged futile treatment.

Patients with myocarditis had the highest survival rate of 64.4% (95% CI 53.9–73.6) in the medical cohort, but also the longest treatment times with a median of 154 hours (IQR 96.5–230). This reproduces the results of similar reports of long support times but good outcomes in case series of patients with myocarditis supported on VA ECMO [28–30]. Similarly, amongst post-surgical patients, those with ECMO after heart transplantation had the highest survival rate at 57.1% (95% CI 49.7–64.2) and were supported for a median of 108 hours (IQR 66–173). These results provide support to other reports of relatively good survival in this post-transplantation group [31–33], and in this large dataset the survival was relatively good despite longer treatment than in other groups.

Furthermore, the lack of any observed interaction between diagnosis and duration of support suggests that the natural history of the disease state treated with ECMO probably influences the baseline hazard rate per time treated, but that the existence of a relationship between time treated and mortality is independent of the indication for support. This may mean that some conditions require shorter or longer periods of time for the eventual outcome to become clinically apparent.

Strengths of this analysis include the very large, international dataset studied, with a contemporary subset taken to reflect “modern” VA ECMO practice. This is larger than any previous analysis of the typical time course of VA ECMO treatment, and has the largest published sample size for adjustment of measured covariates. In addition, treatment time was analysed both in small time quanta (days), and continuously, by estimating the instantaneous hazard function. This preserves as much information about change in mortality rates with time as possible. Other published analysis of treatment duration has categorised this into large groups (for instance, more or less than 2 weeks) [6, 9, 34], or compared only mean or median treatment times in survivors and non-survivors [5, 7].

There are several limitations, principally that while the dataset is large, it reflects voluntary submission of data from a variety of different centres with heterogeneous practice and case-mix. A registry-based dataset has the inherent limitation of reflecting the pooled submitted data of numerous international centres with different patient populations and different treatment practices. While partial adjustment for case-mix can be achieved by examination of baseline measured covariates, these results may not reflect outcomes in any individual unit. Missing data for some baseline covariates were common, restricting the number of covariates that could be included, but similar results for overall effect of treatment duration were seen in the univariate analysis.

Diagnoses were organised into groups, and this would result in some groups being more heterogenous than others, which may limit the applicability of these results to individual indications for ECMO. However, this is a compromise in approach between analysing only pooled data without consideration for diagnosis (which would maximise sample size), and considering only specific indications in isolated analyses (which would restrict the intended broad scope of this study). This study did not attempt to generate a model for individual prognostication, so a moderate number of diagnostic groups were chosen to give broad insights into the different trajectories of treatment between indications.

The non-randomised observational nature of these data limits their applicability in prognostication because selection and reporting bias may influence the distribution of reported cases in the database. For instance, very short runs resulting in treatment failure may be less likely to be reported, particularly if there is inadequate time for data collection, while longer runs may be more likely to be reported. Finally, neurological outcome was not measured in this dataset, so while associations with overall survival were analysed, functional outcome could not be assessed as a secondary outcome.

## Conclusion

Survival in VA ECMO treatment varies with treatment duration, indication for treatment and other patient factors. In the early phase, survival increases per day such that 4 days or less on VA ECMO is associated with a significantly higher mortality rate, most likely reflecting early treatment failure. Survival was observed to be highest when weaned on the fourth day of ECMO but it likely decreases into the second week. Relatively constant survival was observed after this point. While there are survivors of long-term treatment, three quarters of patients receive ECMO for less than one week. Some patients with underlying conditions (like myocarditis and heart transplantation) achieve better outcomes despite longer support duration. While this cannot guide prognostication in individual patients, it is relevant when considering the likely required duration of therapy in different settings. These findings merit further prospective studies for the development of additional prognostic models and weaning strategies.

## Abbreviations

CI: Confidence interval
ECMO: Extracorporeal membrane oxygenation
ELSO: Extracorporeal Life Support Organization
IQR: Interquartile range
OR: Odds ratio
VA ECMO: Veno-arterial extracorporeal membrane oxygenation
VV ECMO: Veno-venous extracorporeal membrane oxygenation
